# Quality of life 10 years after cardiac surgery in adults: a long-term follow-up study

**DOI:** 10.1186/s12955-019-1160-7

**Published:** 2019-05-22

**Authors:** Andrea Perrotti, Fiona Ecarnot, Francesco Monaco, Enrica Dorigo, Palmiero Monteleone, Guillaume Besch, Sidney Chocron

**Affiliations:** 1 Cardio-Thoracic Surgery Department, University Hospital Jean Minjoz, Boulevard Fleming, 25000 Besançon, France; 2 EA 3920, University of Franche-Comté, 25000 Besançon, France; 3 Department of Mental Health, Residential Eating Disorder Unit “Mariconda”, ASL Salerno, Salerno, Italy; 4 Department of Medicine, Surgery and Dentistry “Scuola Medica Salernitana”, Section of Neurosciences, University of Salerno, Salerno, Italy; 5 Department of Anesthesiology and Surgical Intensive Care Unit, University Hospital Jean Minjoz, Boulevard Fleming, 25000 Besançon, France; 6 Department of Cardiology, University Hospital Jean Minjoz, 3, Boulevard Fleming, 25000 Besançon, France

**Keywords:** Quality of life, Cardiac surgery, Follow up, Coronary artery bypass graft

## Abstract

**Background:**

Quality of life (QoL) is a multifactorial concept that assesses physical and mental health. We prospectively studied the quality of life of patients undergoing coronary artery bypass graft (CABG) surgery using the Short-Form 36-item questionnaire (SF-36) up to 10 years after surgery.

**Methods:**

Between January 2000 and December 2002, all patients undergoing elective isolated CABG in the cardiac & thoracic surgery department of a large university hospital in Eastern France underwent initial QoL evaluation with the SF-36. The same questionnaire was mailed to every patient annually (± 2 weeks around the date of surgery) up to 10 years after their operation. We recorded socio-demographic and clinical variables at inclusion. Predictors of impaired QoL at 10 years were identified by logistic regression.

**Results:**

A total of 272 patients (213 men, 59 women) were enrolled; mean age at inclusion was 65 ± 10 years. At 10 years post-surgery, 81 patients had died (29.7%). The physical component summary (PCS) score was significantly higher at 5 years after surgery than at baseline (*p* < 0.01), and significantly lower at 10 years than at 5 years (p < 0.01), although there remained a significant difference between 10-year PCS and baseline score (*p* = 0.004). The mental component summary (MCS) score was significantly higher at 5 years than at the time of surgery (*p* < 0.001), and remained significantly higher compared to baseline at 10 years after surgery (*p* = 0.010). By multivariate analysis, diabetes and dypsnea were both associated with worse PCS at 10 years, while lower age was associated with better 10-year PCS. Only diabetes was associated with impaired MCS at 10 years.

**Conclusions:**

Cardiac surgery appears to durably and positively affect both physical and mental components of quality of life.

**Electronic supplementary material:**

The online version of this article (10.1186/s12955-019-1160-7) contains supplementary material, which is available to authorized users.

## Introduction

The World Health Organization (WHO) has defined health as being “not only the absence of disease and infirmity but also the presence of physical, mental, and social well-being”. To capture this multifaceted concept, quality of life (QoL) has become increasingly important in medicine, social sciences and health care [1, 2], because it reflects not only objective clinical or physiologic status, but more specifically, the patients’ subjective perception about the impact of a clinical condition on their lives, such as the ability to perform physical and social activities, feeling happy in their daily life, and maintain fulfilling interpersonal relationships [3].

One of the main reasons to offer cardiac surgery is to improve both survival and quality of life [4]. In patients whose absolute life expectancy may be limited by age, QoL may therefore be particularly important [5]. There is evidence that the gain in QoL may not be substantial for patients with a low symptom burden at baseline [6], whereas substantial gains in QoL have been reported in patients aged 80 years and over undergoing cardiac surgery [7]. The assessment of QoL prior to cardiac surgery has thus garnered increasing interest among clinicians as a factor to be taken into consideration when estimating the potential benefit to be gained by the patient from the intervention [8]. Indeed, complications such as worsening of psychosocial function may be expected, because patients have to face the challenges of a new life phase that can be accompanied by physical and mental deterioration [9]. It is therefore important for cardiac surgeons to dispose of information about the impact of cardiac surgery on QoL, in order to be able to inform patients apppropriately about the pro and cons of the intervention. The superiority of coronary artery bypass graft surgery (CABG) over a strategy of initial medical therapy in patients with stable coronary artery disease (CAD) or silent ischemia was established in a meta-analysis of seven RCTs over 20 years ago [10] and confirmed in a more recent network meta-analysis [11]. CABG has been shown to be cost-effective at 5 years compared to medical therapy [12], and compared to percutaneous coronary intervention in multivessel CAD [13].

At the time of surgery, several factors have been shown to predict post-operative impairment of QoL, such as age, female sex, history of hypertension, chronic obstructive pulmonary disease, education level, marital status, and also psychological factors such as presence of mood disorders [14]. It has been shown that pre-operative depression is predictive of decreased cardiac symptom relief, quicker recurrence of symptoms, more frequent re-admission, and increased mortality in the immediate post-operative period, while post-operative depression is also associated with poor outcomes such as decreased physical function, increased likelihood of wound infection, increased risk of cardiovascular events and even increased mortality [15, 16]. Therefore, for many patients, maintaining a good QoL is as important as survival [8]. However, there is a lack of data in the literature regarding long-term trends in QoL after CABG surgery, with evaluations often limited to 1 to 2 years after surgery, or rarely, up to a maximum of 5 years [17–19]. We hypothesized that, even if a substantial gain in absolute life expectancy is unlikely for many patients after cardiac surgery, there may be a strong benefit in terms of quality of life over the long-term, which could be an important factor in decision-making. Against this background, our study prospectively analyzed the long-term course of QoL in patients undergoing CABG, through the administration of the Short Form (SF)-36 questionnaire, at baseline (prior to surgery) and then every year up to 10 years after surgery.

## Methods

Between January 2000 and December 2002, all patients scheduled to undergo elective isolated CABG in the department of Cardiac & thoracic surgery of a large University Hospital in Eastern France were invited to undergo QoL evaluation. Patients with cognitive deficit and those who were unable to speak and/or understand French were excluded. Patients provided informed consent and completed the SF-36 questionnaire prior to surgery.

### Outcome measures

The primary outcome measure was QoL, measured using the validated French version of the SF-36 self-assessment questionnaire [20]. The SF-36 is a generic multidimensional instrument consisting of eight domains, namely: physical functioning (PF), role functioning physical (RP); bodily pain (BP); general health perceptions (GH); vitality (VT); social functioning (SF); role functioning emotional (RE) and mental health (MH). Scores are aggregated into two summary measures: the Physical (PCS) and Mental (MCS) Component Summary scores. Scores range from 0 to 100 with higher scores indicating better QoL. PCS high scores indicate no physical limitations, disabilities, or decrements in well-being, as well as high energy level, whereas low scores indicate substantial limitations in self- care, physical, social, and role activities; severe bodily pain or frequent tiredness. MCS high scores indicate frequent positive affect and absence of psychologic distress or limitations in usual social/role activities due to emotional problems, whereas low scores indicate frequent psychologic distress and substantial social and role disability due to emotional problems [21].

The first (pre-operative) assessment was performed after admission, prior to surgery. Then, the questionnaire was mailed to each included patient annually, at a date within 2 weeks (±2 weeks) of the anniversary date of their initial operation. If no reply had been received after one month, another questionnaire was sent. In case of no answer within 2 months, the official records were consulted to obtain the patient’s vital status (dead or alive). If the patient was alive, he/she was considered as a non-responder for the year “N”, but a new questionnaire was sent for year “N + 1”. Only fully completed questionnaires were included in the analysis. There was no imputation of missing data at item-level.

#### Socio-demographic and clinical variables collected

For all patients, we recorded sex, age, marital status, area of residence (rural vs urban), professional activity (in paid employment or not), invalidity status, socio-economic class, level of education (less than high school vs high school diploma and higher), presence of diabetes mellitus, chronic obstructive pulmonary disease (COPD), peripheral artery disease, atrial fibrillation, anxiety and depression (assessed by the Hospital Anxiety and Depression Scale questionnaire), physical disability, angina (assessed by the Canadian Cardiovascular Society score, < 3 vs ≥ 3), and dyspnea (assessed by the New York Heart Association (NYHA) class, < 3 vs ≥ 3). Left ventricular ejection fraction was also measured and is expressed in %. Surgical complications were defined as: periprocedural myocardial infarction or low cardiac output syndrome; mechanical ventilation support for > 24 h or need for reintubation; focal brain injury with permanent or transient deficit; need for dialysis when not previously required or a maximun creatinine serum level more than twice the preoperative value; pneumonia; sepsis with positive cultures; sternal wound infection requiring intravenous antibiotics, surgical debridement, or both; any surgical or invasive treatment consequent to a postoperative adverse event directly associated with the initial cardiac surgery.

Data were obtained from the patient’s medical files.

#### Statistical analysis

Results are expressed as mean ± standard deviation (SD) or number (percentage) for continuous and categorical variables, respectively. Continuous and dichotomous variables were analyzed using the Student t-test, one-way ANOVA, chi-square test or Fisher’s exact test, as appropriate.

Physical Component Summary (PCS) and Mental Component Summary (MCS) scores were constructed. Norm based scoring, where the mean score for the general population is 50 with a standard deviation of 10, is used. Multivariate analysis was performed using logistic regression to predict PCS and MCS at 10 years (separate models for each). Factors with a *p*-value < 0.20 by univariate analysis were included in the multivariate analysis. We also examined the course of QoL scores (PCS and MCS) over the whole duration of follow-up using a mixed model for repeated measures, and Bonferroni correction was applied as appropriate. We performed both univariate and multivariate analyses in the full population, and again in the population of patients who had complete follow-up (i.e. an available questionnaire for pre-operative measurement and for every year of follow-up up to and including 10 years). A *p*-value < 0.05 was considered significant. All statistical analyses were performed using SPSS, Version 18.0 (SPSS, Inc., Chicago, IL, USA).

## Results

Among 585 patients (404 men, 181 women), aged 18 to 89 years old (mean 73 ± 10 years) undergoing scheduled cardiac surgery in our Department during the study period, a total of 272 (46.4%) underwent isolated elective CABG, and were included in the present analysis; 213 (78.3%) were men, mean age was 65 ± 10 years. The characteristics of the study population are shown in Table 1. The perioperative mortality rate was 1.1%. At 10 years, 81 patients had died (29.7%). A total of 73 (26.8%) patients were missing at least one questionnaire. Among these, 38 (52%) failed to return one or more questionnaires, but subsequently returned later questionnaires. The remaining 35 (48%) definitively stopped returning questionnaires during follow-up, and never returned any further questionnaires until the end of follow-up.

**Table 1:** Characteristics of the study population of 272 patients undergoing elective isolated coronary artery bypass graft surgery

Variable	Overall (*N* = 272)	Alive (a) (*N* = 118)	NR & Died (b) (*N* = 154)	*p*-value
				(a) vs (b)
Male	**213 (78)**	**100 (85)**	**113 (73)**	**0.026**
Age	**65 ± 10**	**63 ± 9**	**66 ± 10**	**0.016**
Living maritally	215 (79)	99 (84)	116 (75)	0.057
Living in an urban area	118 (43)	55 (47)	63 (41)	0.39
Professionally active	48 (17)	23 (20)	25 (16)	0.52
On invalidity	7 (3)	4 (3)	3 (2)	0.47
Socio-economic class				
Upper middle class	20 (7)	9 (8)	11 (7)	0.51
Middle class	**52 (19)**	**13 (11)**	**39 (25)**	**0.011**
Lower middle class	66 (24)	37 (31)	29 (19)	0.73
Skilled working class	77 (28)	39 (33)	38 (25)	0.69
Working class	41 (15)	15 (13)	26 (17)	0.81
Others	16 (6)	5 (4)	11 (7)	0.44
Level of education ≥ high school diploma	49 (18)	25 (21)	24 (16)	0.27
Diabetes mellitus	**87 (32)**	**30 (25)**	**57 (43)**	**0.049**
Peripheral arterial disease	67 (25)	24 (20)	43 (36)	0.16
Atrial fibrillation	5 (2)	1 (1)	4 (3)	0.14
Depression	15 (6)	5 (4)	10 (8)	0.59
LVEF				
> 50%	184 (68)	80 (68)	104 (88)	0.78
30–50%	75 (28)	33 (28)	42 (36)	0.93
< 30%	13 (5)	5 (4)	8 (7)	0.67
CCS class ≥3	50 (18)	18 (15)	32 (27)	0.27
NYHA class ≥3	23 (9)	6 (5)	17 (14)	0.12
Complicated Outcome	**38 (14)**	**10 (9)**	**28 (24)**	**0.023**

NR, Non-responders (*N* = 73 patients missing at least one questionnaire at 10 years); Died: *N* = 81 patients had died at 10 years. Data are presented as mean ± standard deviation or number (percentage)

COPD, chronic obstructive pulmonary disease; LVEF, left ventricular ejection fraction, CCS, Canadian Cardiovascular Society; NYHA, New York Heart Association. Variables highlighted in bold show statistically significant differences

### QoL scores at baseline and during the 10-year follow-up period

**Fig. 1:**
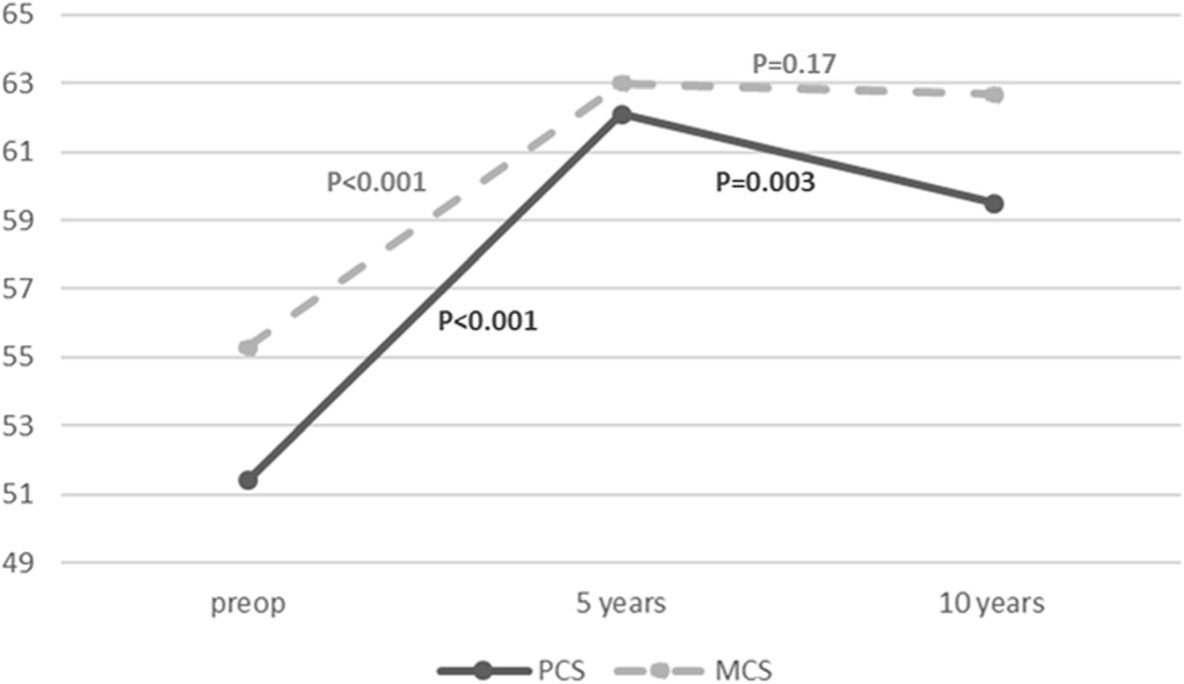
Physical component and mental component summary scores before surgery and over the 10 years of follow-up

The mean raw values of PCS and MCS scores before surgery and at 5 and 10 years of follow-up are given in Table 2 and the course of QoL scores over the 10-year follow-up period is illustrated in Fig. 1. Overall, both component summary scores increased for the first 5 years after surgery, then gradually began to decline, albeit without returning to pre-operative levels. Mean PCS was significantly higher at 5 years than prior to surgery (mean difference 11.3, 95%CI (8.0, 14.6), *p* < 0.001), whereas mean PCS at 10 years deteriorated and was significantly lower than PCS at 5 years (mean difference − 5, 95%CI (− 1.8, − 8.3), *p* = 0.003). Nonetheless, PCS at 10 years remained significantly higher than pre-operative values (*p* = 0.004). Mean MCS was significantly higher at both 5 and 10 years after surgery than pre-operatively (mean difference 7.8, 95%CI (4.6, 11), *p* < 0.001, and 5.8, 95%CI (1.4, 10.2), *p* = 0.010, for MCS at 5 and 10 years respectively vs pre-operative). There was no significant difference between MCS scores at 5 and 10 years (*p* = 0.17).

**Table 2:** Mean Raw Quality of Life Scores prior to surgery, and at 5 and 10 years in 272 patients undergoing elective isolated coronary artery bypass graft surgery

Time	PCS			MCS		
	Mean ± SD	Min	Max	Mean ± SD	Min	Max
Preop	51.4 ± 18.6	10.00	96.25	55.3 ± 20.2	9.6	97.8
5 years	62.1 ± 22.2	8.75	97.5	63.0 ± 20.4	8.6	100.0
10 years	59.5 ± 22.5	13.9	100.0	62.7 ± 20.3	23.1	95.5

PCS, physical component summary score; MCS, mental component summary score of the SF-36

The factors significantly related to PCS and MCS scores at 5 and 10 years by univariate analysis are shown in Table 3. By multivariate logistic regression, younger age was significantly associated with better PCS score at 10 years after surgery (*p* = 0.044), while the presence of diabetes and dyspnea were both associated with significantly worse PCS scores at 10 years (*p* = 0.008 and *p* = 0.011 respectively). Regarding MCS at 10 years, the only factor significantly associated with impaired MCS was diabetes (*p* = 0.007) (Table 4).

**Table 3:** Factors influencing PCS and MCS scores at baseline and at 5 and 10 years post-surgery, by univariate analysis in 272 patients undergoing isolated coronary artery bypass graft surgery

Variable	PCS						MCS					
	Preop		At 5 years		At 10 years		Preop		At 5 years		At 10 years	
	Mean ± SD	*P*-value	Mean ± SD	*P*-value	Mean ± SD	*P*-value	Mean ± SD	*P*-value	Mean ± SD	*P*-value	Mean ± SD	*P*-value
Age												
< 66 years	50.7 ± 18.3	0.51	63.8 ± 23.1	0.21	63.7 ± 21.8	**0.008**	53.6 ± 21.0	0.13	65.1 ± 20.4	0.098	65.9 ± 19.2	**0.029**
> 66 years	52.3 ± 19.1		59.9 ± 21.0		52.4 ± 22.0		57.3 ± 19.0		60.3 ± 20.2		57.3 ± 21.2	
Sexe												
Male	53.3 ± 18.4	**0.002**	63.8 ± 22.4	**0.028**	61.0 ± 22.6	0.072	56.1 ± 19.7	0.21	64.5 ± 20.6	**0.032**	64.4 ± 20.4	**0.023**
Female	44.5 ± 18.0		55.5 ± 20.1		51.1 ± 20.3		52.0 ± 22.0		57.0 ± 18.8		53.3 ± 17.5	
Marital Status												
Alone	49.6 ± 17.9	0.41	58.5 ± 19.0	0.21	57.8 ± 20.0	0.70	56.5 ± 22.6	0.64	61.7 ± 17.1	0.61	59.8 ± 20.0	0.50
Living maritally	51.9 ± 18.8		63.0 ± 22.9		59.8 ± 23.1		54.9 ± 19.6		63.3 ± 21.1		63.3 ± 20.5	
Living in urban area												
No	53.1 ± 19.5	0.20	63.0 ± 23.3	0.63	61.2 ± 20.4	0.42	56.2 ± 21.1	0.51	62.9 ± 20.9	0.97	65.2 ± 19.9	0.22
Yes	50.1 ± 17.9		61.4 ± 21.4		57.9 ± 24.2		54.5 ± 19.6		63.0 ± 20.1		60.6 ± 20.6	
Professional status												
Unemployed	51.9 ± 18.3	0.39	60.3 ± 22.4	**0.005**	57.4 ± 23.1	**0.025**	55.6 ± 19.6	0.65	61.3 ± 20.3	**0.009**	61.2 ± 21.0	0.059
Professionally active	49.1 ± 20.3		71.2 ± 19.0		67.8 ± 18.2		53.9 ± 23.0		71.2 ± 19.2		69.0 ± 16.4	
On invalidity												
Yes	51.6 ± 18.7	0.38	62.9 ± 21.9	0.057	59.8 ± 22.2	0.65	55.3 ± 20.2	0.87	63.3 ± 20.5	0.30	63.0 ± 20.3	0.48
No	45.0 ± 18.2		41.8 ± 23.6		51.2 ± 34.2		53.9 ± 21.2		55.6 ± 17.7		53.4 ± 23.5	
Socio-economic class												
Upper middle class	51.8 ± 20.9	0.22	75.1 ± 15.0	0.14	60.1 ± 26.3	0.82	55.3 ± 26.3	0.77	74.1 ± 14.9	0.33	62.1 ± 24.1	0.93
Middle class	54.1 ± 18.5		59.2 ± 22.5		54.9 ± 22.3		56.5 ± 18.5		61.8 ± 20.1		59.1 ± 20.6	
Lower middle class	54.4 ± 18.6		65.2 ± 22.1		61.1 ± 24.4		56.5 ± 19.6		64.1 ± 20.8		62.7 ± 21.4	
Skilled working class	50.6 ± 18.6		60.4 ± 21.8		61.4 ± 21.0		55.6 ± 20.6		61.6 ± 21.7		65.3 ± 18.9	
Working class	45.9 ± 14.3		59.1 ± 22.9		53.2 ± 21.6		50.7 ± 19.5		61.9 ± 17.0		59.5 ± 20.0	
Others	47.6 ± 24.4		54.5 ± 27.0		61.9 ± 21.5		56.3 ± 22.1		56.1 ± 26.6		62.9 ± 24.6	
Level of education												
< High school	50.8 ± 18.3	0.26	59.6 ± 22.3	**< 0.001**	57.0 ± 22.1	**0.023**	55.0 ± 20.0	0.69	60.7 ± 20.3	**0.001**	60.1 ± 20.1	**0.005**
High school diploma or higher	54.4 ± 19.9		72.7 ± 18.7		68.7 ± 22.0		56.4 ± 21.5		72.9 ± 18.1		72.5 ± 18.5	
Diabetes mellitus												
No	52.2 ± 18.8	0.30	64.3 ± 21.4	**0.033**	61.9 ± 22.0	0.050	55.5 ± 21.5	0.74	63.9 ± 20.5	0.31	65.0 ± 20.2	**0.033**
Yes	49.6 ± 18.2		56.6 ± 23.3		52.3 ± 22.8		54.7 ± 17.2		60.7 ± 20.2		55.9 ± 19.4	
Peripheral arterial disease												
No	52.5 ± 18.9	0.082	63.5 ± 21.3	0.15	60.8 ± 22.3	0.23	56.2 ± 20.8	0.15	64.0 ± 20.0	0.21	63.1 ± 20.6	0.67
Yes	48.0 ± 17.5		57.6 ± 24.7		54.4 ± 23.0		52.2 ± 18.2		59.6 ± 21.5		61.1 ± 19.7	
Depression												
No	51.6 ± 18.6	0.59	62.3 ± 22.4	0.55	59.0 ± 22.3	0.38	55.3 ± 20.3	0.96	63.1 ± 20.5	0.73	62.6 ± 20.4	0.84
Yes	48.8 ± 19.0		58.6 ± 19.5		70.9 ± 26.8		55.0 ± 19.5		61.0 ± 19.1		64.8 ± 22.1	
LVEF												
> 50%	50.4 ± 18.6	0.34	61.3 ± 21.3	0.61	60.1 ± 23.1	0.88	53.9 ± 20.3	0.25	61.9 ± 20.0	0.52	62.6 ± 20.2	0.76
30–50%	52.8 ± 18.0		63.2 ± 24.7		57.8 ± 22.6		57.6 ± 19.8		64.8 ± 21.5		62.0 ± 21.2	
< 30%	57.4 ± 22.0		67.9 ± 21.4		60.1 ± 12.5		61.3 ± 21.6		67.7 ± 19.7		69.3 ± 20.4	
NYHA class ≥3												
No	52.6 ± 18.4	**0.001**	63.0 ± 22.0	0.070	59.5 ± 22.5	0.91	55.8 ± 20.3	0.14	63.8 ± 20.2	0.091	62.6 ± 20.1	0.83
Yes	38.8 ± 16.7		51.0 ± 23.0		58.4 ± 24.4		49.6 ± 18.2		53.5 ± 21.4		65.0 ± 25.3	
CCS class ≥3												
No	53.4 ± 18.4	**< 0.001**	64.0 ± 21.9	**0.011**	60.2 ± 22.0	0.48	55.9 ± 20.1	0.31	64.7 ± 20.0	**0.013**	63.7 ± 19.7	0.29
Yes	42.5 ± 17.2		53.5 ± 21.8		55.6 ± 25.2		52.5 ± 20.5		55.2 ± 20.3		57.3 ± 23.6	
Outcome												
Uneventful	51.2 ± 18.9	0.71	62.7 ± 22.2	0.28	60.3 ± 22.8	0.12	54.9 ± 20.2	0.47	63.3 ± 20.6	0.58	63.4 ± 20.1	0.30
Complicated	52.5 ± 17.0		57.5 ± 21.8		50.2 ± 17.6		57.8 ± 20.6		60.9 ± 19.3		55.3 ± 22.5	

PCS, physical component summary score; MCS, mental component summary score; COPD, chronic obstructive pulmonary disease; LVEF, left ventricular ejection fraction, NYHA, New York Heart Association; CCS, Canadian Cardiovascular Society. Variables highlighted in bold show statistically significant differences

**Table 4:** Factors associated with MCS and PCS at 10 years by multivariate logistic regression^a^ in 272 patients undergoing isolated coronary artery bypass graft surgery

Variable	OR (95%CI)	*P*-value
Physical score (PCS)		
Age	0.95 (0.91–0.99)	0.044
Diabetes	3.72 (1.41–9.75)	0.008
Dyspnea	3.06 (1.29–7.26)	0.011
Mental score (MCS)		
Diabetes	3.46 (1.39–8.59)	0.007

^a^R^2^ = 0.25 (PCS model) and 0.19 (MCS model)

The factors associated with MCS and PCS at 5 and 10 years in the population of 118 patients with complete 10-year follow (i.e. a questionnaire available for every year) by univariate analysis are shown in Additional file 1: Table S1. By multivariate analysis in this population, the factors associated with PCS at 10 years were diabetes and dyspnea, and the sole factor associated with MCS at 10 years was angina (Additional file 1: Table S2).

## Discussion

Our findings indicate that coronary artery bypass graft surgery may have a significant and lasting positive effect on QoL as assessed by the SF36. One of the main goals in proposing CABG surgery is not only the extension of life but rather the improvement of functional mobility, quality of life and maintenance of an independent status [4, 22]. A review concerning QoL benefits after aortic valve surgery in the elderly confirmed that most studies are retrospective and do not compare baseline (pre-surgery) QoL with post-intervention QoL and focus only on patients who survive the follow-up phase [23].

Quality of life, as measured by the SF-36, and its components, is a reflection of the way patients perceive and react to their health status and to non medical aspects of their lives. In our population, both the physical and mental components were observed to improve up to 5 years after surgery, and remained significantly improved at 10 years after surgery, indicating that the patients seem to have yielded a significant benefit from the operation, as perceived and reported via the QoL scores.

The mean PCS score at baseline in our study (51.4 ± 18.6) was similar to that reported in initially healthy individuals from the French SU.VI.MAX cohort, where the mean PCS (measured in 1996) was reported to be 51.1 ± 6.8 [24]. Conversely, our study participants had a mean MCS of 55.3 ± 20.2 at baseline, which is considerably higher than the average of 48.9 ± 8.9 reported from the SU.VI.MAX cohort of initially healthy individuals [24]. This is line with other studies of QoL after surgery showing higher QoL in patients who have had surgery than in the general population [25, 26]. This may be at least partially explained by the response shift phenomenon, whereby individuals revise their standards or priorities with regard to health as they grow older, or if they experience a decline in health [27].

At 5 years after surgery, the mean PCS score observed in our study population was higher than that reported in a population of 140 patients from Poland undergoing CABG, who had a mean PCS of 52.12 at 5 years, compared to 62.1 ± 22.2 in our study [28]. Conversely, the mean MCS at 5 years observed here was the same as in the Polish patients, at 63 in our study, compared to an average of 63.37 in the study by Vincelj et al. [28]. In our study, unmeasured factors may also have contributed to this improvement, such as rehabilitation after the operation, or other supportive care provided in the context of the surgery. Indeed, it has been shown that physiotherapy after CABG surgery has been associated with improvements in QoL [29].

A progressive decline in the PCS score was observed between 5 and 10 years after surgery, indicating that the disease may have started to progress again, causing discomfort and functional impairment to the patients, and this is reflected in the physical component of their QoL scores. Indeed, it has been shown that mortality risk in patients undergoing isolated CABG is similar to, or only slightly higher than that of the general population up to 10 years, but increases thereafter due to progression of underlying disease or potential loss of graft patency [30, 31].

Conversely, in our study, MCS maintained a significant improvement over baseline up to 10 years after surgery. This is line with the Polish study of health-related QoL in 140 patients undergoing CABG surgery, who were found to have higher MCS at 5 years than PCS. These authors concluded that physical limitations persist after CABG that affect physical functioning, but do not affect emotional and mental functioning as much [28]. The discrepancy in the trajectory of MCS and PCS may also be due to natural aging, which may affect physical and mental capacities differently [32, 33]. Indeed, it has been shown that mental and physical health are state-dependent, and past physical health reportedly has stronger effects on present mental health than health investments, income or education [32]. In a cohort study of over 40,000 individuals from Northern Norway, Lorem et al. found that ageing had a negative effect on self-reported health, with a strong association between mental health symptoms and physical disease [33]. These authors concluded that the effect on self-reported health of mental health symptoms caused by physical illness is an increasing public health problem [33]. In line with these observations, continued follow-up of the cohort from our study would allow us to assess whether the mental component scores also begin to follow the same trajectory as physical decline.

Regarding the interaction between chronic diseases and QoL outcomes of individuals undergoing cardiac surgery, we found that diabetes and dyspnea were significantly associated with worse QoL at 10 years in the overall population, while in those with full follow-up, angina was also found to be related with worse MCS at 10 years. These findings are coherent with those of Rumsfeld et al. who reported that peripheral vascular disease, chronic obstructive pulmonary disease (COPD), arterial hypertension and low left ventricular ejection fraction predicted lower follow-up PCS [23]. In our sample, diabetes and dyspnea were associated with lower QoL score at 10 years, although one might expect surgery to have provided some relief of dyspnea symptoms. However, the persistence of this symptom could be suggestive of heart failure, which over the long term would have a deleterious effect on physical symptoms as it became more pronounced. In the population with full follow-up, the persistence of angina could similarly be a source of discomfort, and limit the capacity to remain socially active, which is a determinant of the mental health dimension of the SF36. This effect may also be partially mediated by depression, although this factor was not signifiant by multivariate analysis in our study. Indeed, several authors have previously identified mental distress as a high risk factor for impaired QoL after cardiac surgery. Depression (or depressive symptoms) impairs patients’ perceptions of their QoL within the psychosocial domain. Postoperative depression has been shown to be associated with decreased physical function, increased risk of cardiovascular events (in particular angina), increased likelihood of wound infection and increased mortality [16]. The combined presence of depression and angina has been shown to be quite consistent across countries and continents [34] and this link deserves further exploration in our population, as it might indirectly explain (at least partially) the finding that angina was associated with impaired MCS scores at 10 years in our study.

Some limitations must be taken into consideration when interpreting the results of our study. As the present study was conducted at a single institution, generalization of the results might be limited and institutional bias may be present in relation to patient selection and postoperative management. In addition, this was not a randomized controlled trial, so some confounding may persist. Since patient selection criteria and the spectrum of preoperative morbid conditions frequently vary across studies, it is difficult to make reliable comparisons [22]. Missing data and patient attrition also need to be considered. At 10 years, a total of 73 patients had at least one missing questionnaire, of whom 35 had definitively stopped returning questionnaires. We cannot exclude the possibility that these patients stopped returning the questionnaires for reasons related to poor health and/or poor QoL. The level of missing data at item level was practically nil and therefore unlikely to affect the robustness of the analyses. Thirdly, although all patients from our centre are systematically referred to the same cardiac rehabilitation centre, we cannot exclude the possibility that different rehabilitation conditions, or other unmeasured confounders, may have affected QoL. Fourth, the SF36 is a generic QoL questionnaire, and not disease-specific, although it has been reported that it is sensitive to the detection of changes in health-related quality of life variables in patients with coronary artery disease after CABG surgery [35].

## Conclusions

Reliable information on the impact of cardiac surgery on QoL is important for both patients and surgeons, to help them weigh the benefits and risks of the intervention. Our study shows that the physical component of QoL improves significantly up to 5 years after surgery, but may begin to decline thereafter, while the mental component of QoL is durably positively affected by coronary artery bypass graft surgery.

## Additional file

Additional file 1: **Table S1**. Factors influencing PCS and MCS scores at baseline and at 5 and 10 years post-surgery, by univariate analysis in 118 patients undergoing isolated coronary artery bypass graft surgery with complete follow-up (i.e. Quality of Life questionnaire available every year up to 10 years post-surgery). **Table S2**. Factors associated with PCS and MCS scores at 10 years post-surgery by Logistic Regression Analysis in 118 patients undergoing isolated coronary artery bypass graft surgery with complete follow-up (i.e. Quality of Life questionnaire available every year up to 10 years post-surgery). (DOCX 34 kb)

## Abbreviations

BP: Bodily Pain
CABG: Coronary Artery Bypass Grafting
CCS: Canadian Cardiovascular Society scale
COPD: Chronic Obstructive Pulmonary Disease
GH: General Health
HRQoL: Health-Related Quality of life
ICU: Intensive Care Unit
MCS: Mental Component Summary score
MH: Mental Health
NBS: Norm Based Scoring
PCS: Physical Component Summary score
PF: Physical Functioning
QoL: Quality of Life
RE: Role functioning Emotional
RP: Role functioning Physical
RR: Risk Ratio
SD: Standard Deviation
SF: Social Functioning
SF-36: Short Form 36
SMR: Standardized Mortality Ratio
VT: Vitality
