# Supplementary material for: Quality of life 10 years after cardiac surgery in adults: a long-term follow-up study
Source: Health Qual Life Outcomes. 2019 May 22;17:88. doi: 10.1186/s12955-019-1160-7 (PMC6532216; doi:10.1186/s12955-019-1160-7)
Supplement: Supplementary file 1 — Table S1. Factors influencing PCS and MCS scores at baseline and at 5 and 10 years post-surgery, by univariate analysis in 118 patients undergoing isolated coronary artery bypass graft surgery with complete follow-up (i.e. Quality of Life questionnaire available every year up to 10 years post-surgery). Table S2. Factors associated with PCS and MCS scores at 10 years post-surgery by Logistic Regression Analysis in 118 patients undergoing isolated coronary artery bypass graft surgery with complete follow-up (i.e. Quality of Life questionnaire available every year up to 10 years post-surgery). (DOCX 34 kb) [file 12955_2019_1160_MOESM1_ESM.docx]

**Additional file 1: Table S1:** Factors influencing PCS and MCS scores at baseline and at 5 and 10 years post-surgery, by univariate analysis in 118 patients undergoing isolated coronary artery bypass graft surgery with complete follow-up (i.e. Quality of Life questionnaire available every year up to 10 years post-surgery).

| Variable | **PCS** | | | | | | | **MCS** | | | | | | | | | | | |
| --- | --- | --- | --- | --- | --- | --- | --- | --- | --- | --- | --- | --- | --- | --- | --- | --- | --- | --- | --- |
|  | Preop | | At 5years | | At 10 years | | | Preop | | | | At 5years | | | | At 10 years | | | |
|  | Mean ± SD | P-value | Mean ± SD | P-value | Mean ± SD | | P-value | | Mean ± SD | | P-value | | Mean ± SD | | P-value | | Mean ± SD | | P-value |
| **Age** | | | | | | | | | | | | | | | | | | | |
| < 66 years | 41.7 ± 7.4 | 0.71 | 45.3 ± 9.6 | 0.43 | 43.8 ± 9.4 | **0.010** | | 43.2 ± 10.7 | | 0.47 | | 47.2 ± 8.9 | | 0.55 | | 47.2 ± 8.6 | | 0.20 | |
| > 66 years | 41.1 ± 7.7 |  | 44.1 ± 7.7 |  | 39.2 ± 9.2 |  |  | 44.6 ± 9.7 | |  |  | 46.1 ± 9.6 | |  |  | 44.8 ± 10.3 | |  |  |
| **Sexe** | | | | | | | | | | | | | | | | | | | |
| Male | 42.1 ± 6.8 | **0.037** | 45.0 ± 9.1 | 0.63 | 42.6 ± 9.7 | 0.15 | | 43.3 ± 10.2 | | 0.39 | | 47.4 ± 8.6 | | 0.19 | | 47.1 ± 9.3 | | **0.022** | |
| Female | 38.1 ± 10.1 |  | 44.0 ± 8.3 |  | 39.4 ± 8.2 |  |  | 45.8 ± 11.0 | |  |  | 43.6 ± 11.3 | |  |  | 41.8 ± 8.3 | |  |  |
| **Marital Status** | | | | | | | | | | | | | | | | | | | |
| Alone | 39.9 ± 8.0 | 0.31 | 43.4 ± 8.1 | 0.42 | 40.8 ± 8.1 | 0.50 | | 44.3 ± 12.6 | | 0.78 | | 46.7 ± 8.9 | | 0.95 | | 44.2 ± 10.3 | | 0.28 | |
| Living maritally | 41.8 ± 7.4 |  | 45.1 ± 9.1 |  | 42.4 ± 9.8 |  |  | 43.6 ± 9.8 | |  |  | 46.8 ± 9.2 | |  |  | 46.7 ± 9.1 | |  |  |
| **Living in urban area** | | | | | | | | | | | | | | | | | | | |
| No | 41.2 ± 7.6 | 0.72 | 44.4 ± 9.0 | 0.55 | 42.1 ± 10.4 | 0.97 | | 43.5 ± 10.4 | | 0.83 | | 46.5 ± 9.5 | | 0.67 | | 45.3 ± 9.3 | | 0.22 | |
| Yes | 41.7 ± 7.3 |  | 45.4 ± 8.9 |  | 42.1 ± 8.5 |  |  | 43.9 ± 10.3 | |  |  | 47.2 ± 8.7 | |  |  | 47.4 ± 9.3 | |  |  |
| **Professional status** | | | | | | | | | | | | | | | | | | | |
| Unemployed | 41.3 ± 7.7 | 0.57 | 44.1 ± 9.1 | 0.055 | 41.3 ± 9.8 | 0.074 | | 43.7 ± 10.2 | | 0.92 | | 46.3 ± 9.0 | | 0.18 | | 45.8 ± 9.7 | | 0.25 | |
| Professionally active | 42.3 ± 6.5 |  | 48.0 ± 7.5 |  | 45.3 ± 7.7 |  |  | 43.9 ± 11.2 | |  |  | 49.1 ± 9.3 | |  |  | 48.3 ± 7.2 | |  |  |
| **On invalidity** | | | | | | | | | | | | | | | | | | | |
| Yes | 41.2 ± 6.8 | 0.93 | 30.2 ± 8.4 | **0.034** | 39.2 ± 16.5 | 0.74 | | 35.2 ± 10.3 | | 0.18 | | 40.6 ± 6.2 | | 0.12 | | 42.8 ± 8.2 | | 0.45 | |
| No | 41.5 ± 7.5 |  | 45.4 ± 8.5 |  | 42.2 ± 9.3 |  |  | 44.0 ± 10.2 | |  |  | 47.0 ± 9.2 | |  |  | 46.4 ± 9.4 | |  |  |
| **Socio-economic class** | | | | | | | | | | | | | | | | | | | |
| Upper middle class | 41.3 ±9.8 | 0.79 | 47.3 ± 9.1 | 0.80 | 41.8 ± 11.3 | 0.76 | | 47.0 ± 14.9 | | 0.22 | | 50.4 ± 8.9 | | 0.69 | | 45.1 ± 10.6 | | 0.9 | |
| Middle class | 42.6 ± 6.2 |  | 45.0 ± 7.0 |  | 40.9 ± 8.8 |  |  | 39.3 ± 10.0 | |  |  | 46.8 ± 11.4 | |  |  | 46.1 ± 8.6 | |  |  |
| Lower middle class | 42.2 ± 7.6 |  | 45.0 ± 10.2 |  | 43.2 ± 10.6 |  |  | 44.0 ± 9.3 | |  |  | 46.1 ± 8.9 | |  |  | 46.0 ± 9.7 | |  |  |
| Skilled working class | 41.6 ± 6.7 |  | 45.2 ± 8.2 |  | 42.6 ± 8.8 |  |  | 45.8 ± 10.0 | |  |  | 47.8 ± 8.9 | |  |  | 47.6 ± 8.4 | |  |  |
| Working class | 39.0 ± 6.0 |  | 41.9 ± 9.6 |  | 38.9 ± 9.3 |  |  | 40.2 ± 10.7 | |  |  | 44.8 ± 8.8 | |  |  | 45.2 ± 10.2 | |  |  |
| Others | 40.3 ± 14.5 |  | 44.5 ± 3.4 |  | 43.2 ± 7.8 |  |  | 41.1 ± 7.5 | |  |  | 44.6 ± 8.6 | |  |  | 43.6 ± 12.6 | |  |  |
| **Level of education** | | | | | | | | | | | | | | | | | | | |
| < High school | 40.9 ± 7.2 | 0.19 | 43.8 ± 9.3 | **0.003** | 41.3 ± 9.5 | 0.064 | | 43.5 ± 10.2 | | 0.74 | | 50.3 ± 9.3 | | **0.041** | | 45.3 ± 9.3 | | **0.030** | |
| High school diploma or higher | 43.4 ± 8.4 |  | 48.7 ± 6.3 |  | 45.2 ± 9.1 |  |  | 44.4 ± 11.0 | |  |  | 45.9 ± 8.9 | |  |  | 49.8 ± 8.7 | |  |  |
| **Diabetes mellitus** | | | | | | | | | | | | | | | | | | | |
| No | 41.4 ± 6.9 | 0.93 | 43.6 ± 9.8 | 0.39 | 39.3 ± 10.5 | 0.084 | | 43.6 ± 8.2 | | 0.92 | | 47.0 ± 8.6 | | 0.91 | | 44.4 ± 9.6 | | 0.21 | |
| Yes | 41.5 ± 7.7 |  | 45.3 ± 8.6 |  | 43.1 ± 9.0 |  |  | 43.8 ± 11.0 | |  |  | 46.8 ± 9.3 | |  |  | 46.9 ± 9.2 | |  |  |
| **Peripheral arterial disease** | | | | | | | | | | | | | | | | | | | |
| No | 42.6 ± 6.2 | 0.34 | 41.7 ± 10.5 | 0.087 | 39.6 ± 9.8 | 0.16 | | 40.5 ± 10.6 | | 0.092 | | 45.2 ± 9.5 | | 0.34 | | 46.6 ± 8.4 | | 0.81 | |
| Yes | 41.2 ± 7.8 |  | 45.7 ± 8.3 |  | 42.8 ± 9.4 |  |  | 44.6 ± 10.1 | |  |  | 47.2 ± 9.0 | |  |  | 46.2 ± 9.6 | |  |  |
| **Depression** | | | | | | | | | | | | | | | | | | | |
| No | 41.6 ± 7.3 | 0.50 | 45.0 ± 8.9 | 0.46 | 41.9 ± 9.4 | 0.35 | | 43.5 ± 10.2 | | 0.38 | | 46.9 ± 9.2 | | 0.63 | | 46.4 ± 9.4 | | 0.54 | |
| Yes | 38.3 ± 10.0 |  | 41.6 ± 9.1 |  | 47.5 ± 11.9 |  |  | 49.2 ± 12.9 | |  |  | 44.9 ± 8.5 | |  |  | 43.6 ± 8.9 | |  |  |
| **LVEF** | | | | | | | | | | | | | | | | | | | |
| >50% | 41.3 ± 7.6 | 0.73 | 45.0 ± 8.6 | 0.91 | 42.9 ± 9.7 | 0.42 | | 43.6 ± 11.0 | | 0.96 | | 46.4 ± 9.3 | | 0.80 | | 45.9 ± 9.1 | | 0.57 | |
| 30-50% | 42.1 ± 7.3 |  | 44.7 ± 9.7 |  | 40.4 ± 9.6 |  |  | 44.1 ± 8.7 | |  |  | 47.6 ± 8.7 | |  |  | 46.5 ± 9.7 | |  |  |
| <30% | 39.5 ± 7.5 |  | 43.3 ± 9.6 |  | 40.6 ± 4.6 |  |  | 43.1 ± 11.9 | |  |  | 47.9 ± 9.6 | |  |  | 50.4 ± 11.1 | |  |  |
| **NYHA class ≥ 3** | | | | | | | | | | | | | | | | | | | |
| No | 41.8 ± 7.3 | **0.023** | 45.0 ± 9.0 | 0.22 | 42.2 ± 9.5 | 0.57 | | 44.0 ± 10.3 | | 0.13 | | 46.9 ± 9.2 | | 0.47 | | 46.2 ± 9.1 | | 0.74 | |
| Yes | 34.8 ± 7.4 |  | 41.5 ± 6.1 |  | 39.7 ± 10.0 |  |  | 37.7 ± 8.4 | |  |  | 44.4 ± 7.7 | |  |  | 48.0 ± 12.9 | |  |  |
| **CCS class ≥ 3** | | | | | | | | | | | | | | | | | | | |
| No | 42.3 ± 7.4 | **0.004** | 45.2 ± 9.0 | 0.32 | 42.3 ± 9.3 | 0.54 | | 44.0 ± 10.9 | | 0.31 | | 47.4 ± 9.0 | | 0.12 | | 46.9 ± 42.7 | | 0.076 | |
| Yes | 37.0 ± 6.3 |  | 42.9 ± 8.6 |  | 40.7 ± 10.6 |  |  | 42.1 ± 6.4 | |  |  | 43.6 ± 9.2 | |  |  | 42.7 ± 10.5 | |  |  |
| **Outcome** | | | | | | | | | | | | | | | | | | | |
| Uneventful | 41.7 ± 7.5 | 0.38 | 45.1 ± 8.9 | 0.27 | 42.5 ± 9.7 | **0.038** | | 43.2 ± 9.9 | | 0.24 | | 46.8 ± 9.0 | | 0.92 | | 46.4 ± 9.2 | | 0.68 | |
| Complicated | 39.4 ± 7.5 |  | 41.8 ± 8.5 |  | 37.5 ± 6.2 |  |  | 48.8 ± 13.8 | |  |  | 46.5 ± 10.6 | |  |  | 44.8 ± 11.4 | |  |  |

**Additional file 1: Table S2:**  Factors associated with PCS and MCS scores at 10 years post-surgery by Logistic Regression Analysis in 118 patients undergoing isolated coronary artery bypass graft surgery with complete follow-up (i.e. Quality of Life questionnaire available every year up to 10 years post-surgery).

| ***Variable*** | **Odd Ratio** | **95.0% Confidence Interval** | | **p-Value** |
| --- | --- | --- | --- | --- |
|  |  | **Lower Bound** | **Upper Bound** |  |
| *Physical score* | | | | |
| Diabetes | 4.21 | 1.67 | 8.20 | 0.048 |
| Dyspnea | 3.32 | 1.47 | 6.28 | 0.002 |
| *Mental score* | | | | |
| Angina | 2.57 | 1.35 | 3.21 | 0.018 |
